# Urinary and serum levels of high mobility group box 1

**DOI:** 10.1016/j.plabm.2023.e00327

**Published:** 2023-07-11

**Authors:** Toshihiko Nakamura, Atsushi Yoshida, Daisuke Hatanaka, Michiko Kusakari, Hidehiro Takahashi, Shinji Katsuragi, Shingo Yamada, Takashi Kamohara

**Affiliations:** a Department of Neonatology, Japanese Musashino Red Cross Hospital, Japan; b Department of Obstetrics and Gynecology, Sakakibara Heart Institute, Japan; c Shino-test Coroporation, Japan

**Keywords:** ELISA, Acetylated truncated HMGB-1, Urinary, Serum, Neonates, Hypoxic ischemic encephalopathy

## Abstract

**Background:**

High mobility group box 1 (HMGB-1) has been extensively studied in adults and to a certain extent in neonates as well. Clinical examination of neonates, especially unwell neonates soon after birth, should be minimally invasive.

**Objective:**

This study aimed to investigate whether the urinary HMGB-1 level is comparable to the serum HMGB-1 level in neonates.

**Methods:**

In all, 87 neonates (37.5 ± 2.9 weeks of gestation and a mean birth weight of 2588 ± 649 g) were enrolled. Of these, 53 were males and 34 were females. The umbilical cord blood and the first or second spontaneous voiding urine samples were stored, and the HMGB-1 level in the samples was measured.

**Results:**

HMGB-1 was detected in all urinary samples. In these samples, we found acetylated HMGB-1 and may be devoid of nine residues at the N-terminal amino acid sequence. There was a significant correlation between the serum HMGB-1 level and urinary HMGB-1 level (r = 0.73, p < 0.001). Urinary HMGB1 levels in fetal neonatal asphyxia were significantly higher than those in healthy controls (p = 0.09).

**Conclusion:**

Urinary excretion may be one of the metabolic pathways of HMGB-1. The urinary HMGB-1 level may be comparable to the serum HMGB-1 level in the early neonatal period.

## Highlights

• Urinary HMGB-1 is acetylated.
• There was a significant correlation between the serum HMGB-1 level and the urinary HMGB-1 level.

## Abbreviations

HMGB-1: high mobility group box 1 DAMP: damage-associated molecular pattern
HIF: hypoxic ischemic encephalopathy β2-MG: β2 microglobulin
α1-MG: α1microglobulin SLE: systemic lupus erythematosus
ANCA-AVV: anti-neutrophil ctoplasmic antibody-associated vasculitis

## 1 Introduction

High mobility group box 1 (HMGB-1) was recently found to function as a damage-associated molecular pattern (DAMP) when released passively from either dead, dying/injured cells or secreted by immune/cancer cells in response to endogenous and/or exogenous stimuli, such as hypoxia, endotoxin etc. [[1], [2], [3]].

HMGB-1 acts as an important proinflammatory mediator and is capable of activating inflammation and tissue repair. HMGB-1 has been extensively studied in adult patients [4,5] and to a certain extent in neonates as well [[6], [7], [8], [9], [10]].

In this study, we aimed to examine whether urinary HMGB-1 level is comparable to serum HMGB-1 and can thus avoid invasive blood collection in all neonates. This is the first report about urinary HMGB-1 in neonates.

## 2 Methods and material

The study subjects were 87 neonates delivered at the Department of Obstetrics, Perinatal Center at the Japanese Red Cross Musashino Hospital and at Sakakibara Heart Institute, during July 2017 to June 2018, and November 2017 to June 2018, respectively. All neonates’ parents provided written informed consent.

Serum was separated from the umbilical cord blood samples within 1 h after birth and stored at -80 °C. Spontaneously voided urine samples were collected using urine bags attached to the supra-pubic abdominal skin soon after birth.

Fusion was carried out according to the method of Milstein [11]. We selected that the monoclonal antibody was capable to react with the peptide Gly11-Ala17 (GKMSSYA), but not with the peptide Lys7-Ala17(KKPRGKMSSYA). The cells selected were subcloned until their monoclonality was demonstrated. Antibodies are purified by an affinity purification method using Protein A.

Electrophoresis and Immunoblotting were performed. The samples were subjected to 15% SDS-PAGE gel electrophoresis. Anti-acetyl lysine polyclonal antibody (Cell signal Lab. USA) or monoclonal antibodies to an HMGB1 degradation product beginning from the Gly11 of HMGB-1 (Asahi Kasei Pharma Corp, Japan) were used. Peroxidase-labeled anti-Rabbit Ig polyclonal antibody (Dako, Denmark) or peroxidase-labeled anti-Mouse Ig polyclonal antibody (Dako, Denmark) was diluted 5000-fold with PBS containing 3% BSA. The nitrocellulose membrane was soaked in reaction reagent ECL (GE Health Science, USA) according to the manufacturer's instructions. Chemiluminescence-labeled bands were visualized using Lumino Graph Ⅲ WSE 6300 (ATTO Co. Japan). HMGB1 was determined by immunoblot analysis and quantified from the blots by measurement of the absorbance of the band (NIH 1.59 software; NIH) and comparison with a calibration curve constructed with purified human HMGB1 serially diluted in normal human serum.

The anti-human HMGB1 monoclonal antibodies and anti-HMGB-1peptide polyconal antibodies against the peptide sequence with different from the sequence of HMGB2 were developed, and the antibodies were used to construct sandwich ELISA methods with a chromogenic substrate (TMBZ) [12]. Serum and Urinary HMGB1 levels were measured using this assay [12].

StatMate™ version Ⅳ (StatMate, Atms, Tokyo, Japan) was used to evaluate the results. Parameters are expressed as means and standard deviations (SD). Correlations between the serum and urinary concentrations of HMGB-1 were analyzed using the Spearman rank correlation test. Receiver operating characteristic (ROC) curve analysis was used to evaluate the sensitivity and specificity of urinary HMGB-1. Differences were considered statistically significant at p<0.05.

## 3 Results

**Fig. 1:**
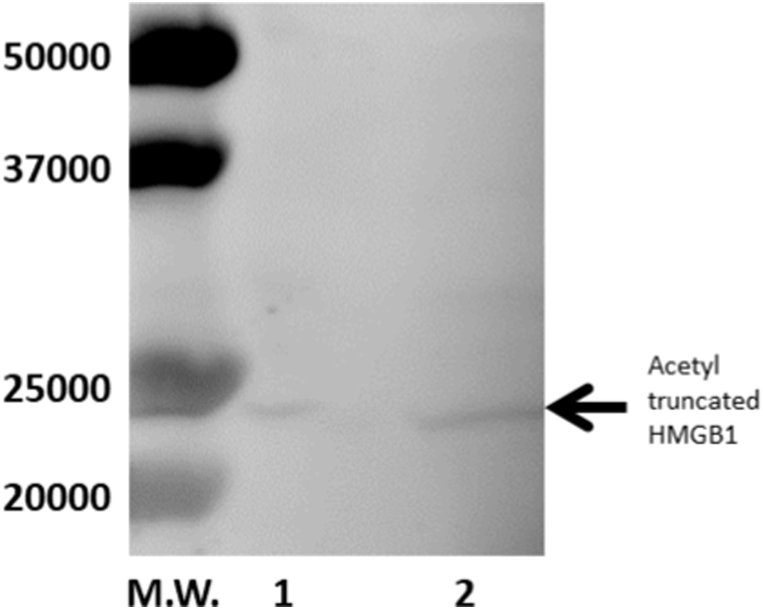
High mobility group box 1 (HMGB1) concentrations in urine from neonates. lane 1,2: urine samples of neonates lane 1: The nitrocellulose membrane was reacted with anti-acetyl lysine polyclonat antibody. lane 2: The nitrocellulose membrane was reacted with monoclonal antibodies against cleavage amino acid sequence Arg10-Gly11(R-G) of the N-terminal domain.

Using urinary samples, the detection limit of HMGB-1 was achieved at a concentration >0.2 ng/mL. The intra-assay ranged from 4.1% to 7.3% and the inter-assay ranged from 4.8% to 8.5%. Recovery, calculated from the data (n = 10) of several concentrations of purified human HMGB-1 added to the pooled urine, as the ratio of the observed concentration to the expected concentration multiplied by 100%, ranged from 92 to 111% in the HMGB-1 ELISA assay [13]. The results obtained from urinary samples were normalized to urinary creatinine (as per creatinine clearance concentration, μg/g.Cr). The urinary level of creatinine was analyzed using the creatinine picrate reaction. We measured HMGB1 levels in urinary samples from five neonates at Japanese Red Cross Musashino Hospital using western blotting and ELISA. The correlation between the values obtained using both methods was good. The urinary reference value in neonatal ranged from 9.9 to 264.3 ng/mL (N = 31). In neonates, urinary HMGB1 was acetylated and appeared to be metabolized because it had a molecular weight of approximately 25 KDa (Fig. 1). Furthermore, this urinary HMGB-1 reacted with monoclonal antibodies against cleavage amino acid sequence Arg10-Gly11(R-G) of the N-terminal domain.

**Fig. 2:**
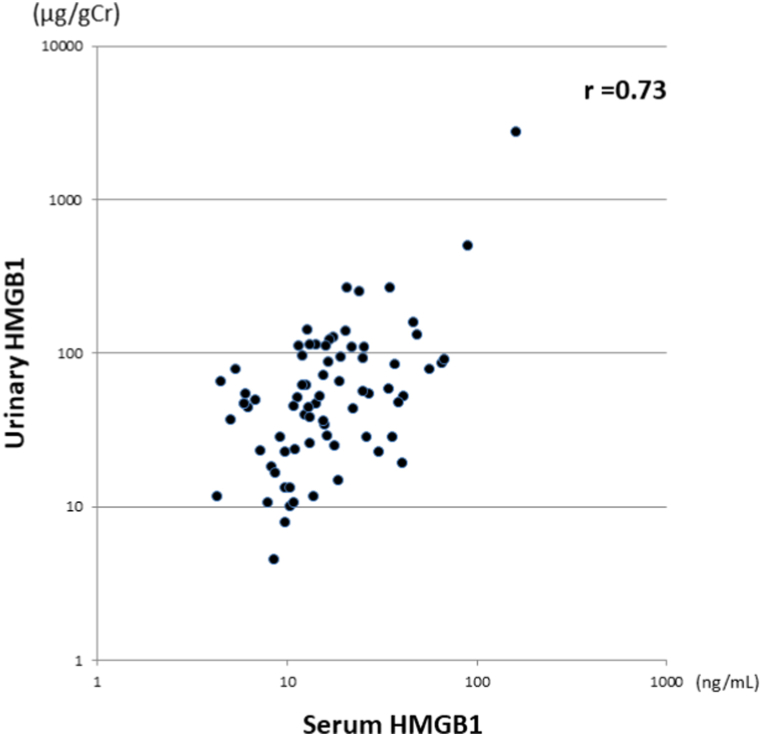
Correlation between serum HMGB-1 level and urinary HMGB-1 level There was a significant correlation between the serum and the urinary HMGB-1 (r = 0.77, p<0.001). Both axes of the graph are logarithmically scaled.

The 87 subjects were included in this analysis. The mean gestational age was 37.5 ± 2.9 weeks, and the mean birth weight was 2588 ± 649 g. The cohort comprised 53 males and 34 females; 38 were transvaginal deliveries, 27 were scheduled cesarean sections, and 22 were unscheduled cesarean sections. There was a significant correlation between the serum HMGB-1 level and the urinary HMGB-1 level (r = 0.73, p < 0.001) (Fig. 2). On ROC analysis of the urinary HMGB-1 level for predicting abnormally high serum HMGB-1, the area under the curve (AUC) for urinary HMGB-1 was 0.70052, and the urinary HMGB-1 cut-off of 70 μg/g.Cr identified neonates with serum high HMGB-1 levels, with a sensitivity of 60.0% and specificity of 70.8% (p < 0.001). Urinary HMGB1 levels in neonatal asphyxia were significantly higher than those in healthy controls (p = 0.09). The same was found for serum HMGB1 levels (P = 0.06). The urinary concentration of HMGB-1 in 17 infants whose levels were serially measured, significantly decreased exponentially (p < 0.005).

## 4 Discussion

Urinary HMGB1 of neonatal seemed to be degraded because it had a molecular weight of approximately 25 KDa. In addition, urinary HMGB-1 reacted with monoclonal antibodies against the cleavage amino acid sequence Arg10-Gly11(R-G) of the N-terminal domain of HMGB-1. Ito et al. reported that thrombin decomposes HMGB-1 with a molecular weight of 25 KDa [14]. Furthermore, because the monoclonal antibody specific to the cleavage site showed a band at a similar position, this 25 KDa HMGB-1 may be the residual HMGB-1 devoid of nine residues at the N-terminal domain. Ito et al. also reported that the residual HMGB-1 devoid of nine residues at the N-terminal domain exhibited no toxicity [14]. In other words, it is highly possible that the HMGB-1 that we observed was a thrombin-modified and detoxified metabolite of HMGB-1 that was actively secreted from the cell. This result is considered an example of HMGB1 metabolism.

HMGB-1 is a low molecular weight (LMW) protein with a size similar to a LMW protein of 40 kDa or less, such as molecular weight 30 KDa, β2-microglobulin (β2-MG) 11.8 kDa, and α1-microglobulin (α1-MG) 33 kDa. These urinary LMW proteins normally pass freely through the glomeruli, and most of them are reabsorbed by the renal tubules; hence, only trace amounts are detected in the urine. Since β2-MG and HMGB-1 are almost similar in molecular weight, we hypothesized that an increase in the blood HMGB-1 level would be reflected in the urinary HMGB-1 level. Until now, no study focused on urinary HMGB-1 has investigated whether it could be substituted for serum HMGB-1. However, three types of renal impairment complications have been found in systemic lupus erythematosus (SLE) patients [15,16], namely, calcium nephrolithiasis [17], nephritis in anti-neutrophil cytoplasmic antibody (ANCA)-associated vasculitis (AAV) [18,19], and the ischemia reperfusion model of the kidney [20]. The common factor in these three complications and the ischemia-reperfusion model of renal implantation is the research design, which assumes that HMGB-1 excreted in the urine can be measured from the locally injured kidney tissue. The purpose of our study was to investigate whether the HMGB-1 observed in the blood is excreted in the urine without resorption from the kidneys of the newborn as a low molecular protein, not kidney injury. The difference between HMGB-1 and β2-MG is the difference in their blood and urine concentrations. Although the HMGB-1 levels in the blood are not high in such patients, the corresponding HMGB-1 level was detected in the urine, and a strong positive correlation was seen in 87 subjects. This correlation has not been discussed in the studies on β2-MG. According to this study, HMGB-1 was detected in the urine in the early neonatal period, and its urinary concentration reflected the serum concentration. A sensitivity of 60% and a specificity of 70.8% (p<0.001) were obtained when the cut-off value of urinary concentration was 70 μg/g.Cr. However, this study did not include subjects with pathological states causing high serum HMGB-1, such as asphyxia [13,14], severe infection [5], and fetal inflammatory response syndrome [21].

## 5 Conclusions

Urinary excretion may be one of the metabolic pathways of HMGB-1. The urinary HMGB-1 level may be comparable to the serum HMGB-1 level in the early neonatal period.

## Author contribution

Toshihiko Nakamura: Dr Nakamura conceptualized and designed the study, drafted the initial manuscript, and approved the final manuscript as submitted.

Atsushi Yoshida: Dr Yoshida conceptualized and designed the study, drafted the initial manuscript.

Daisuke Hatanaka: Dr Hatanaka conceptualized and designed the study, drafted the initial manuscript.

Michiko Kusakari: Dr Kusakari conceptualized and designed the study, drafted the initial manuscript.

Hidehiro Takahashi: Dr Takahashi conceptualized and designed the study, drafted the initial manuscript.

Shinji Katsuragi: Dr Katsuragi conceptualized and designed the study, drafted the initial manuscript.

Shingo Yamada: Mr Yamada measured serum concentrations of HMGB-1, reviewed and revised the manuscript, and approved the final manuscript as submitted.

Takashi Kamohara: Dr Kamohara conceptualized and designed the study, drafted the initial manuscript, and approved the final manuscript as submitted.

## Financial of disclosure

The authors have no financial relationships relevant to this article to disclose.

There are no prior publications or submissions with any overlapping information, including studies and patients.

## Declaration of competing interest

The authors declare that they have no known competing financial interests or personal relationships that could have appeared to influence the work reported in this paper.

There is no other.

## Data Availability

The data that has been used is confidential.
